# Effectiveness of REGEN-COV antibody combination in preventing severe COVID-19 outcomes

**DOI:** 10.1038/s41467-022-32253-9

**Published:** 2022-08-02

**Authors:** Samah Hayek, Yatir Ben-shlomo, Noa Dagan, Ben Y. Reis, Noam Barda, Eldad Kepten, Alina Roitman, Shachar Shapira, Shlomit Yaron, Ran D. Balicer, Doron Netzer, Alon Peretz

**Affiliations:** 1 Clalit Research Institute, Innovation Division, Clalit Health Services, Tel Aviv, Israel; 2 Software and Information Systems Engineering, Ben Gurion University, Be’er Sheva, Israel; 3 Department of Biomedical Informatics, Harvard Medical School, Boston, MA USA; 4 The Ivan and Francesca Berkowitz Family Living Laboratory Collaboration at Harvard Medical School and Clalit Research Institute, Boston, MA USA; 5 Predictive Medicine Group, Computational Health Informatics Program, Boston Children’s Hospital, Boston, MA USA; 6 Harvard Medical School, Boston, MA USA; 7 Clalit Community Division, Clalit Health Services, Tel Aviv, Israel; 8 Israel Defense Forces Medical Corps, Tel Aviv, Israel; 9 Department of Military Medicine, Faculty of Medicine, Hebrew University, Jerusalem, Israel; 10 School of Public Health, Faculty of Health Sciences, Ben Gurion University of the Negev, Be’er Sheva, Israel

**Keywords:** Epidemiology, SARS-CoV-2, Viral infection, Drug development

## Abstract

REGEN-COV, a combination of the monoclonal antibodies casirivimab and imdevimab, has been approved as a treatment for high-risk patients infected with SARS-CoV-2 within five days of their diagnosis. We performed a retrospective cohort study, and used data repositories of Israel’s largest healthcare organization to determine the real-world effectiveness of REGEN-COV treatment against COVID-19-related hospitalization, severe disease, and death. We compared patients infected with Delta variant and treated with REGEN-COV (n = 289) to those infected but not-treated with REGEN-COV (n = 1,296). Demographic and clinical characteristics were used to match patients and for further adjustment as part of the C0x model. Estimated treatment effectiveness was defined as one minus the hazard ratio. Treatment effectiveness of REGEN-COV was 56.4% (95% CI: 23.7–75.1%) in preventing COVID-19 hospitalization, 59.2% (95% CI: 19.9–79.2%) in preventing severe COVID-19, and 93.5% (95% CI: 52.1–99.1%) in preventing COVID-19 death in the 28 days after treatment. In conclusion, REGEN-COV was effective in reducing the risk of severe sequelae in high-risk COVID-19 patients.

## Abstract (web summary)

REGEN-COV is a SARS-CoV-2 combined monoclonal antibody treatment which has been shown to be effective in randomised controlled trials. Here, the authors assess its real-world effectiveness using data from Israel during the Delta wave and find that it reduced the risk of hospitalisation, severe disease and death.

## Introduction

Since December 2019, SARS-CoV-2 has spread worldwide^1^, resulting over 310 million confirmed infections and until roughly over 5.4 million deaths up to January 2022^2^. SARS-CoV-2 vaccines, first introduced in December 2020, have helped reduce the burden of the pandemic. Yet, there remain many people who are not vaccinated, either due to lack of vaccine access, prior medical conditions, or vaccine hesitancy^3^. In addition, breakthrough infections occur among vaccinated individuals^4^. Therefore, there is still a need for effective treatments to prevent severe COVID-19 outcomes among infected individuals.

REGEN-COV, a combination of two SARS-CoV-2 antibodies (casirivimab and imdevimab), received emergency use authorization from the U.S. Food and Drug Administration for patients aged 12 years or older in 2021. The drug is designated for patients who have been diagnosed with COVID-19 and have not deteriorated to severe illness, but are at high risk for deterioration, including immunocompromised individuals and those with high-risk medical conditions^5^. The efficacy of REGEN-COV has been demonstrated in clinical trials^5–7^, where it was shown to reduce symptomatic COVID-19 illness by 62% and COVID-19-related hospitalization or death by 70% in the first month after treatment^5^. Evidence suggests that the benefits are greater when REGEN-COV is administered within the first five days following infection^8, 9^.

An observational study by Razonable et al., 2022^10^, which includes 696 patients who received REGEN-COV, was compared with propensity-matched controls of 696 non-treated patients diagnosed with mild to moderate COVID-19 at Mayo clinic sites in four US states. The results indicate that the REGEN-COV treatment significantly lowered all-cause hospitalization rates at day 14 (1.3% vs 3.3%; absolute difference[AD]: 2.0%; 95% confidence interval (CI): 0.5–3.7%) at day 21 (1.3% vs 4.2%; AD: 2.9%; 95% CI: 1.2–4.7%), and at day 28 (1.6% vs. 4.8%; AD: 3.2%; 95% CI: 1.4–5.1%). The effectiveness of REGEN-COV against all-cause ICU admission rates and all-cause mortality at three-time points were similar in both groups^10^. This study has specific limitations as it also presents non-COVID-19 outcomes. This might limit the ability to infer the effectiveness of REGEN-COV against hospitalizations, ICU admissions, and mortality due to COVID-19. Thus, there is a need to further explore the effectiveness of REGEN-COV in health outcomes related to COVID-19, from a massive nationwide intervention setting.

The Israeli Ministry of Health began a nationwide campaign to administer REGEN-COV at a dose of 1200 mg on September 22, 2021, during a period in which the Delta variant was the dominant variant in Israel, and Israel’s fourth wave of COVID-19 was at its peak. As part of this campaign, Clalit Health Services (CHS), Israel’s largest healthcare organization, proactively offered REGEN-COV treatment at home to eligible members who were diagnosed with COVID-19. Members were eligible if they were within 5 days of their first positive Polymerase Chain Reaction (PCR) test and were determined to be at high risk for severe illness, but had not yet developed severe COVID-19. The determination of risk was made by clinicians at the point of care, based on patient’s overall likelihood of deterioration to severe illness. The likelihood of deterioration to severe illness was evaluated using a simple score-based model that was developed by Clalit Health Services and adopted by the Israeli Ministry of Health^11^.

The present study aims to evaluate the real-world effectiveness of REGEN-COV treatment in preventing COVID-19-related hospitalization, severe illness, and death. A retrospective cohort study was conducted using the data repositories of Israel’s largest healthcare organization. Patients diagnosed with COVID-19 between September 19, 2021, and December 8, 2021, who were treated with REGEN-COV were matched to patients diagnosed with COVID-19 between July 1, 2021, and December 8, 2021, who were not treated with REGEN-COV. Analysis was performed using Cox regression, with estimated treatment effectiveness defined as one minus the hazard ratio. A subgroup analysis was conducted as a secondary analysis by age group (<60 or ≥60 years old).

## Results

In total, 162,795 CHS members tested positive for COVID-19 between July 1 and December 8, 2021. Of this population, 306 were enrolled in our cohort and treated with REGEN-COV between September 19, 2021, and December 8, 2021, and 162,489 were non-treated with REGEN-COV. Exclusion criteria for both treated and non-treated with REGEN-COV included: participants aged <12 years old (*n* = 2610); participants without continuous CHS membership (n = 3562); participants who previously had the omicron variant (*n* = 37); participant data that had invalid information related to COVID-19 (e.g., death date, hospitalization date) (*n* = 514); participants hospitalized before the index date(*n* = 4); missing data on participant smoking status (*n* = 1750), and missing data on BMI (*n* = 18,857). 135,458 were eligible for inclusion in the study, and patients were matched on a 1:5 ratio. Of those, 149 controls were excluded before contributing follow-up time as they developed one of the outcomes before their treated match could receive the treatment. The final analysis included 289 patients treated with REGEN-COV and 1296 non-treated matched patients (Supplemental Fig. 1).

The median age of the population was 67 years (interquartile range: 58–74 years), and 48% were men. Patients treated with REGEN-COV were more likely to be smokers compared to those who were non-treated (14% vs. 11%, respectively) (Table 1). All matched variables were well-balanced after matching between the treated and non-treated groups (Supplemental Fig. 2). The difference between those who were treated with REGEN-COV to non-treated participants (potential controls) is described in Supplemental Table 1. Furthermore, Supplemental Table 1 describes the difference between those who were unmatched and non-treated individuals to matched individuals. The unmatched and non-treated individuals were younger and had fewer chronic conditions.

**Table 1:** Baseline characteristics of the study population, by REGEN-COV status

	Total participants (*N* = 1583)	Non-treated participants (*N* = 1296)	Treated with REGEN-COV (*N* = 289)
Age [Mean(SD), median (IQR)]	65 (14), 67 (58,74)	65 (14), 67 (58,74)	66 (14), 68 (58,76)
**Age group, in years**			
19–29	12 (0.8%)	10 (0.8%)	2 (0.7%)
30–39	51 (3.2%)	44 (3.4%)	7 (2.4%)
40–49	192 (12%)	157 (12%)	34 (12%)
50–59	195 (12%)	163 (13%)	33 (11%)
60–69	475 (30%)	390 (30%)	86 (30%)
70–74	266 (17%)	221 (17%)	44 (15%)
75+	392 (25%)	311 (24%)	83 (29%)
**Population sector**			
General Jewish	1133 (72%)	929 (72%)	205 (71%)
Arab	347 (22%)	282 (22%)	66 (23%)
Orthodox Jewish	103 (6.5%)	85 (6.6%)	18 (6.2%)
**Sex**			
Female	819 (52%)	671 (52%)	149 (52%)
Male	764 (48%)	625 (48%)	140 (48%)
**Socioeconomic status**			
Low	1078 (68%)	890 (69%)	189 (65%)
Medium	468 (30%)	383 (30%)	86 (30%)
High	36 (2.3%)	22 (1.7%)	14 (4.8%)
Missing	1 (<0.1%)	1 (<0.1%)	0 (0%)
**Flu vaccination in the last five years**			
0	422 (27%)	368 (28%)	55 (19%)
1	207 (13%)	168 (13%)	39 (13%)
2	180 (11%)	148 (11%)	32 (11%)
3	165 (10%)	131 (10%)	34 (12%)
4	213 (13%)	155 (12%)	58 (20%)
5	396 (25%)	326 (25%)	71 (25%)
**Body mass index (kg/m**^**2**^**)**			
Normal	372 (23%)	66 (23.0%)	306 (24.0%)
Obese	508 (32%)	126 (44.0%)	561 (43.0%)
Overweight	687 (43%)	92 (32.0%)	416 (32.0%)
Underweight	18 (1.1%)	5 (1.7%)	13 (1.0%)
**Smoking status**			
Current smoker	184 (12%)	141 (11%)	41 (14%)
Past smoker	393 (25%)	318 (25%)	75 (26%)
Non-smoker	1006 (64%)	837 (65%)	173 (60%)
Recent full vaccination	1187 (75%)	1010 (78%)	176 (61%)
**First vaccination dose**			
Unvaccinated	513 (32%)	415 (32%)	99 (34%)
0–3 weeks	692 (44%)	573 (44%)	121 (42%)
4–7 weeks	190 (12%)	162 (12%)	27 (9.3%)
8–10 weeks	121 (7.6%)	102 (7.9%)	19 (6.6%)
11–19 weeks	57 (3.6%)	39 (3.0%)	18 (6.2%)
≥20 weeks	10 (0.6%)	5 (0.4%)	5 (1.7%)
**Chronic conditions**			
Cancer	84 (5.3%)	59 (4.6%)	25 (8.7%)
Chronic kidney disease	313 (20%)	227 (18%)	87 (30%)
Respiratory diseases	254 (16.1%)	199 (15.4%)	57 (19.7%)
Cardiovascular disease	475 (30%)	233 (18%)	116 (41.0%)
Pregnancy	11 (0.7%)	9 (0.7%)	2 (0.7%)
Diabetes	552 (34.9%)	427 (32.9%)	125 (43.1%)
Hypertension	731 (46%)	573 (44%)	158 (55%)
Immunosuppression	91 (5.7%)	74 (5.7%)	17 (5.9%)
Neurological disease	180 (11%)	143 (11%)	38 (13%)
Liver disease	72 (4.5%)	51 (3.9%)	21 (7.3%)

Abbreviations: *IQR* interquartile range.

As compard with non-treated patients, among those treated with REGEN-COV the risk of hospitalization due to COVID-19 decreased by 56.4% (95% CI: 23.7–75.1%); the risk of severe COVID-19 illness decreased by 59.2% (95% CI: 19.9–79.2%); and the risk of COVID-19-related death decreased by 93.5% (95% CI: 52.1–99.1%) (Table 2). A full description of the Cox model, which describes this information, is presented in Supplemental Table 2−4.

**Table 2:** Outcomes associated with REGEN-COV treatment effectiveness

		Received REGEN-COV (*n* = 289)	Did not received REGEN-COV (*n* = 1296)	Unadjusted REGEN-COV effectiveness (95%CI)	Adjusted REGEN-COV effectiveness (95% CI)
Hospitalization due to COVID-19	Yes	15	105	36.8% (−8.0–63.2%)	56.4% (23.7–75.1%)
Severe COVID-19	Yes	10	82	46.0% (−4.2–72.0 %)	59.2% (19.9–79.2%)
Death due to COVID-19	Yes	1	27	83.3% (−23.0–97.7%)	93.5% (52.1–99.1%)

Abbreviation: *CI* Confidence Interval.

Note: Treatment effectiveness was measured as a 1-Hazard ratio (HR), derived from a Cox–proportional model that was applied after the matching. Patients were matched using an optimal matching scheme, including the following variables: Age, population sector, sex, SES, BMI, immunosuppression status, pregnancy, and first vaccination dose status.

The Cox model was then further adjusted for age, population sector, sex, SES, BMI, number of flu vaccines received in the five years prior to COVID-19 infection, smoking status, recent full vaccination status, first vaccination dose, and chronic diseases (cancer, chronic kidney disease, respiratory diseases, cardiovascular diseases, diabetes, hypertension, immunosuppression, neurological conditions, and liver diseases). Complete variable definitions are found in Supplemental Table 7.

The results of the secondary analysis showed that among those aged 60 years or older and treated with REGEN-COV, the risk of hospitalization due to COVID-19 decreased by 57.0% (95% CI: 16.0–75.7%); the risk of severe COVID-19 illness decreased by 61.1% (95% CI: 21.0–76.4%); and the risk of COVID-19-related death decreased by 94.4% (95% CI: 58.8–99.2%). Among those younger than 60 years old, the risk of hospitalization due to COVID-19 decreased by 91.5% (95% CI: 28.2–99.0%). However, due to the rarity of severe COVID-19 and death in this age group, the effectiveness of REGEN-COV for these outcomes could not be accurately estimated (Supplemental Table 5).

The sensitivity analysis results using propensity score matching confirm the main analysis and indicate that REGEN-COV effectively reduces the risk of severe COVID-19 hospitalization due to COVID-19 and mortality due to COVID-19 (Supplemental Table 6).

## Discussion

In the current study, we estimated the effectiveness of community-based REGEN-COV treatment for patients newly infected with SARS-CoV-2 (Delta variant) who were determined to be at high risk for severe COVID-19, but who had not yet developed severe disease. Our results indicate that treatment with REGEN-COV was effective in reducing the risk of hospitalization due to COVID-19, severe COVID-19, and COVID-19-related death among patients overall and specifically for those aged 60 years or older.

The results of this real-world study are consistent with the results of the phase-III clinical trial, which showed that treatment with REGEN-COV reduced the risk of hospitalization or death by 70.4% in the 28 days following treatment initiation^12^. They are also consistent with the results of an observational study that showed a 70% reduction in the need for further treatment among those treated with REGEN-COV^9^. Importantly, the effectiveness of REGEN-COV has recently been reported to be diminished against the Omicron variant, which has become dominant in many regions worldwide.

The current study had several limitations. First, despite the careful matching of treated and non-treated individuals and the further adjustment in the Cox regression, there exists the possibility of residual confounding, specifically by behavioral factors that were not well-captured in our data. Second, the recruitment of the non-treated patients started 10-weeks earlier than the treated patients, which could result in confounding by calendar time. However, the circumstances in Israel were not different between the first 10-weeks and the remaining study period, so we do not expect this to result in substantial bias. Third, there is the possibility of selection bias among those treated with REGEN-COV compared to those who refused to be treated with REGEN-COV and were included as controls. However, only a small number of the control population is composed of individuals who refused treatment, which limits the potential bias. Fourth, REGEN-COV is less effective against the Omicron variant; the infectiousness and severity of Omicron were three times higher compared to the original Wuhan strain^13^. Finally, the number of treated participants is relatively small and the number of outcomes is small which lead to imprecise estimates.

This is the first study to uniquely characterize the effectiveness of REGEN-COV from real-world data that is based on a nationwide cohort enrollment from a massive treatment campaign, showing the effectiveness of a national-scale intervention using antivirals. The cohort was based on a large representative cohort that covers over half of the Israeli population, providing novel evidence regarding the effectiveness of a national treatment campaign in which treatment was offered equally to all eligible patients. The study results reveal high effectiveness of REGEN-COV in treating high-risk COVID-19 patients recently infected with the Delta variant. With many individuals remaining unvaccinated and breakthrough infections occurring among the vaccinated, effective treatment for COVID-19 remains vital.

## Methods

### Data sources

Data was extracted from the CHS database. CHS is the largest integrated payer-provider healthcare organization in Israel. The CHS database contains extensive medical histories of CHS’s 4.7 million members, including COVID-19 test results and outcomes. These data repositories have been previously described in detail^14, 15^.

### Population and study design

We conducted a cohort study using CHS data to estimate the real-world effectiveness of REGEN-COV in preventing severe COVID-19-related outcomes. Eligibility criteria included: a documented first positive SARS-CoV-2 polymerase-chain-reaction (PCR) test result; a determination of being high risk for severe COVID-19 based on medical history and clinical characteristics; age 12 years-old or older; and at least one year of continuous CHS membership as of the infection date. We excluded patients who were known to be infected with the Omicron variant. We assessed Omicron infections based on sequencing of viral samples or the S-gene target failure (SGTF) technique; the prevalence of Omicron during the study period was negligible. We also excluded patients with invalid outcome data (e.g. invalid hospitalization data, etc) and those who received a positive PCR result during hospitalization for another condition.

To emulate a target trial, treated patients were individually matched with non-treated patients. Treated patients were those with a first positive PCR test result obtained between September 19, 2021 and December 8, 2021 and who received REGEN-COV treatment: Non-treated patients were those who obtained a first positive PCR test result between July 1, 2021 and December 8, 2021 and who did not receive REGEN-COV treatment. The recruitment period for the non-treated patients was a few weeks longer than for the treated patients in order to increase the sample size of the non-treated group and allow for a 1:5 matching of treated to non-treated individuals. Matching was performed using an optimal matching scheme. The Mahalanobis distance metric was used for continuous variables, and exact matching was used for categorical variables^16, 17^. Optimal matching minimizes the overall pairwise distances without dependency on the order of matching.

REGEN-COV was indicated but not provided to certain high-risk individuals for a range of possible reasons. These reasons included: the patient was diagnosed in the weeks before REGEN-COV was being offered by the healthcare system; logistic complexity prevented the distribution of the treatment to the patient’s home; or the patient refused to receive the treatment.

### Outcomes and Follow-up

Three outcomes were examined: 1) COVID-19-related hospitalization, 2) severe COVID-19 illness, and 3) death due to COVID-19, all the outcomes were defined as per the Israeli Ministry of Health. The treated and non-treated patients were followed from the assigned index date. The index date for the treated patients was defined as the date of REGEN-COV treatment. Non-treated and matched patients were given an index date based on the relative time from infection diagnosis to treatment of the matched treated patient. For example, if the treated patient received REGEN-COV two days after their positive PCR test result, the index date for the matched non-treated patient was set to two days after their positive PCR test result. Follow-up ended with the occurrence of the outcome or at 28-days following the index date.

### Covariates adjustment

Adjustments were performed in two phases. First, the treated and non-treated patients were matched on an initial set of potential confounders. Covariates included in the analysis were based on the input of domain experts and the scientific literature related to the factors associated with the COVID-19 health outcomes and the likelihood of receiving REGEN-COV^18, 19^.

Subjects were matched based on: age; sex; population sector (Jewish, Arab, Ultra-Orthodox); socioeconomic status (SES, based on place of residence and categorized into 20 levels); body mass index (BMI as a continuous variable); immunosuppression status; pregnancy; and calendar week of first vaccination dose.

Further adjustment was done using a Cox Proportional Hazards Regression. Confounders that were adjusted for in the model included: age; sex; population sector, SES. (as stated above); BMI (as a categorical variable: underweight, normal and obese); the number of flu vaccines received within the five years prior to COVID-19 diagnosis; smoking status; recent full vaccination status; and calendar week of first vaccination dose. We also adjusted for the chronic conditions (e.g. cardiovascular disease, diabetes, respiratory disease, are hypertension) described in Supplemental Table 7. The regression included some of the variables that were used in matching to better control possible residual confounding, for reasons further detailed under the statistical analysis section.

All variables were extracted according to the most recently documented value before the positive testing date, as recorded in the patients’ medical records. Full variable definitions are presented in Supplemental Table 7.

Matched non-treated individuals who experienced an outcome between their positive PCR test date and their assigned index date were excluded. Because the index date was only set after matching (based on the matched treated counterpart’s timing), this exclusion could only happen after matching.

### Statistical analysis

Following matching, Cox proportional hazards models were fit for each outcome, adjusting for the abovementioned potential confounders. We performed a collinearity test and excluded the number of vaccination doses received due to high collinearity with a recent full vaccination.

We report one minus the hazard ratio (HR) with 95% confidence intervals as the measure of treatment effectiveness. The Cox modeling included some variables that were already used for the matching process for two reasons. First, some of the variables were continuous, and the matching was not exact. Second, not all the treated subjects had the same number of controls due to the exclusion of controls post-matching.

Missing data are rare occurrence in CHS database for the variables used; thus, we used a complete case analysis.

A subgroup analysis by age group (<60 or ≥60 year old) was conducted as a secondary analysis.

### Sensitivity analysis

To assess the sensitivity of our results to the matching method, we repeated the analysis with a different matching approach - Propensity Score Matching (PSM). Multivariable logistic regression was conducted to generate propensity scores. The propensity score model included age, population sector, sex, SES, BMI, immunosuppression status, pregnancy, and first vaccination dose status as a surrogate for health-seeking behavior. For each treated subject, we matched a non-treated subject, based on propensity score similarity (1:5 matching ratio).

### Ethics

This study was approved by the CHS Institutional Review Board (0052-20-COM2).

### Reporting summary

Further information on research design is available in the Nature Research Reporting Summary linked to this article.

## Supplementary information

Supplementary Information

Reporting Summary

## Data Availability

Due to national and organizational data privacy regulations, individual-level data such as those used for this study cannot be shared. Access to the data used for this study can be made available upon request, subject to an internal review to ensure that participant privacy is protected, and subject to completion of a data sharing agreement, approval from the institutional review board of CHS and institutional guidelines and in accordance with the current data sharing guidelines of CHS and Israeli law. Pending the aforementioned approvals, data sharing will be made in a secure setting, on a per-case-specific manner, as defined by the chief information security officer of CHS.

## Code availability

The analytic code is available at https://github.com/clalitresearch/Regeneron_Effectiveness.
