# Supplementary material for: Effectiveness of REGEN-COV antibody combination in preventing severe COVID-19 outcomes
Source: Nat Commun. 2022 Aug 2;13:4480. doi: 10.1038/s41467-022-32253-9 (PMC9344792; doi:10.1038/s41467-022-32253-9)

Effectiveness of REGEN-COV Antibody Combination in Preventing Severe COVID-19 Outcomes

Samah Hayek[1], Yatir Ben-shlomo[1], Noa Dagan[1,2,3,4], Ben Y. Reis[4,5,6], Noam Barda[2,3], Eldad Kepten[1], Alina Roitman[7], Shachar Shapira[8,9], Shlomit Yaron[7], Ran D.Balicer[1,4,10,,11], Doron-Netzer[7,11], Alon Peretz[7,11]

Affiliation

[1] Clalit Research Institute, Innovation Division, Clalit Health Services, Tel Aviv, Israel.
[2] Software and Information Systems Engineering, Ben Gurion University, Be'er Sheva, Israel.
[3] Department of Biomedical Informatics, Harvard Medical School, Boston, MA, USA.
[4] The Ivan and Francesca Berkowitz Family Living Laboratory Collaboration at Harvard Medical School and Clalit Research Institute, Boston, MA, USA.
[5] Predictive Medicine Group, Computational Health Informatics Program, Boston Children's Hospital, Boston, MA, USA.
[6] Harvard Medical School, Boston, MA, USA.
[7] Clalit Community Division, Clalit Health Services, Tel Aviv, Israel.
[8] Israel Defense Forces Medical Corps, Tel Aviv, Israel.
[9] Department of Military Medicine, Faculty of Medicine, Hebrew University, Jerusalem, Israel.
[10] School of Public Health, Faculty of Health Sciences, Ben Gurion University of the Negev, Be'er Sheva, Israel.
[11] These authors contributed equally: Ran.D. Balicer, Doron Netzer, Alon Peretz

Corresponding author:
Samah Hayek, DrPH
Clalit Research Institute
Toval 40, Ramat Gan, Israel
samahha@clalit.org.il
T: +972-3-6925809

Contents

Supplemental Table 1: Baseline characteristics of the eligible comparison population (non-treated participants) by matching status……………………………………………………………………3

Supplemental Table 2: Cox proportional hazard model for the association between REGEN-COV and risk for Severe COVID-19 ……………………………………………………………………………6

Supplemental Table 3: Cox proportional hazard model for the association between REGEN-COV and risk for hospitalization……………………………………………………………………………9

Supplemental Table 4: Cox proportional hazard model for the association between REGEN-COV and risk for COVID-19 related death…………………………………………………………………12

Supplemental Table 5: Outcomes associated with REGEN-COV treatment effectiveness, by age grop……………………………………………………………………………………………………15

Supplemental Table 6: Outcomes associated with REGEN-COV treatment effectiveness using a PSM approach…………………………………………………………………………………………16

Supplemental Table 7: Variable definitions…………………………………………………………17

Supplemental Figure legends…………………………………………………………………………24

Supplemental Figure 1: Flow chart of study participants……………………………………………25

Supplemental Figure 2: Love plot ……………………………………………………………………25

Supplemental Table 1: Baseline characteristics of the eligible comparison population (non-treated participants) by matching status

	Treated with REGEN-COV (N=289)	Total non-treated participants (N=135,169) (Potential controls)	Unmatched non-treated participants (N=133,873)	Matched non-treated participants (N=1,296)
Age [Mean(SD), median(IQR)]	66 (14), 68 (58,76)	38 (18), 36 (24,48)	38 (18), 35 (24,47)	65 (14), 67 (58,74)
Age group, in years				
**12-18**	-	22,735 (17%)	22,735 (17%)	0 (0%)
**19-29**	2 (0.7%)	25,628 (19%)	25,618 (19%)	10 (0.8%)
**30-39**	7 (2.4%)	31,728 (23%)	31,684 (24%)	44 (3.4%)
**40-49**	34 (12%)	24,080 (18%)	23,923 (18%)	157 (12%)
**50-59**	33 (11%)	12,574 (9.3%)	12,411 (9.3%)	163 (13%)
**60-69**	86 (30%)	9,654 (7.1%)	9,264 (6.9%)	390 (30%)
**70-74**	44 (15%)	3,369 (2.5%)	3,148 (2.4%)	221 (17%)
**75+**	83 (29%)	5,401 (4.0%)	5,090 (3.8%)	311 (24%)
Population sector				
**General Jewish**	205 (71%)	95,946 (71%)	95,017 (71%)	929 (72%)
**Arab**	66 (23%)	29,737 (22%)	29,455 (22%)	282 (22%)
**Orthodox Jewish**	18 (6.2%)	9,477 (7.0%)	9,392 (7.0%)	85 (6.6%)
**Not reported**	0(0%)	9 (<0.1%)	9 (<0.1%)	0 (0%)
Sex				
**Female**	149 (52%)	76,250 (56%)	75,579 (56%)	671 (52%)
**Male**	140 (48%)	58,919 (44%)	58,294 (44%)	625 (48%)
Socioeconomic status				
**Low**	189 (65%)	88,408 (65%)	87,518 (65%)	890 (69%)
**Medium**	86 (30%)	43,440 (32%)	43,057 (32%)	383 (30%)

**High**	14 (4.8%)	3,198 (2.4%)	3,176 (2.4%)	22 (1.7%)
**Missing**	0 (0%)	123 (<0.1%)	122 (<0.1%)	1 (<0.1%)
Flu vaccination in the last five years				
**0**	55 (19%)	83,671 (62%)	83,303 (62%)	368 (28%)
**1**	39 (13%)	19,645 (15%)	19,477 (15%)	168 (13%)
**2**	32 (11%)	10,300 (7.6%)	10,152 (7.6%)	148 (11%)
**3**	34 (12%)	6,804 (5.0%)	6,673 (5.0%)	131 (10%)
**4**	58 (20%)	5,729 (4.2%)	5,574 (4.2%)	155 (12%)
**5**	71 (25%)	9,020 (6.7%)	8,694 (6.5%)	326 (25%)
Body mass index (kg/m^2)				
**Underweight**	13(1.0%)	61,189 (45%)	60,883 (45%)	306 (24%)
**Normal**	306(24.0%)	28,198 (21%)	27,637 (21%)	561 (43%)
**Obese**	561(43.0%)	40,322 (30%)	39,906 (30%)	416 (32%)
**Overweight**	416(32.0%)	5,460 (4.0%)	5,447 (4.1%)	13 (1.0%)
Smoking status				
**Current smoker**	41 (14%)	17,848 (13%)	17,707 (13%)	141 (11%)
**Past smoker**	75 (26%)	18,635 (14%)	18,317 (14%)	318 (25%)
**Non-smoker**	173 (60%)	98,686 (73%)	97,849 (73%)	837 (65%)
Recent full vaccination	176 (61%)	113,711 (84%)	112,701 (84%)	1,010 (78%)
First vaccination dose				
**Unvaccinated**	99 (34%)	41,301 (31%)	40,886 (31%)	415 (32%)
**0-3 weeks**	121 (42%)	27,623 (20%)	27,050 (20%)	573 (44%)
**4-7 weeks**	27 (9.3%)	27,093 (20%)	26,931 (20%)	162 (12%)
**8-10 weeks**	19 (6.6%)	24,962 (18%)	24,860 (19%)	102 (7.9%)
**11-19 weeks**	18 (6.2%)	8,635 (6.4%)	8,596 (6.4%)	39 (3.0%)
**≥20 weeks**	5 (1.7%)	5,555 (4.1%)	5,550 (4.1%)	5 (0.4%)
Chronic conditions				

**Cancer**	25 (8.7%)	1,862 (1.4%)	1,803 (1.3%)	59 (4.6%)
**Chronic kidney disease**	87 (30%)	8,369 (6.2%)	8,142 (6.1%)	227 (18%)
**Respiratory diseases**	57 (19.7%)	13,103(9.7%)	12,904(9.7%)	199(15.4%)
**Cardiovascular disease**	116 (41.0%)	5,639 (4.2%)	5,406 (4.0%)	233 (18%)
**Pregnancy**	2 (0.7%)	4,741 (3.5%)	4,732 (3.5%)	9 (0.7%)
**Diabetes**	125 (43.1%)	11,240(8.3%)	10,813(8.1%)	427(32.9%)
**Hypertension**	158 (55%)	14,402 (11%)	13,829 (10%)	573 (44%)
**Immunosuppression**	17 (5.9%)	1,224 (0.9%)	1,150 (0.9%)	74 (5.7%)
**Neurological disease**	38 (13%)	5,909 (4.4%)	5,766 (4.3%)	143 (11%)
**Liver disease**	21 (7.3%)	2,500 (1.8%)	2,449 (1.8%)	51 (3.9%)

Abbreviations: IQR, interquartile range.

Supplemental Table 2: Cox proportional hazard model for the association between REGEN-COV and risk for Severe COVID-19

Covariates	Categories	HR	Standard error	Confidence interval (lower)	Confidence interval (upper)	P-value
**REGEN-COV unadjusted**	Yes vs. no	0.54	0.335	0.28	1.042	0.066
**REGEN-COV adjusted**	Yes vs. no	0.408	0.344	0.208	0.801	0.009
**Age**	Continuous	1.038	0.012	1.013	1.064	0.002
**Number of flu vaccination in the last five years**	1 versus 0	1.070	0.425	0.465	2.462	0.874
	2 versus 0	0.929	0.445	0.388	2.225	0.869
	3 versus 0	1.128	0.437	0.480	2.656	0.782
	4 versus 0	0.697	0.467	0.279	1.742	0.440
	5 versus 0	0.671	0.444	0.281	1.603	0.370
**Obesity**	Obese versus normal	2.509	0.330	1.314	4.789	0.005
	Overweight versus normal	1.567	0.341	0.804	3.055	0.188
	Underweight versus normal	0.676	1.094	0.079	5.768	0.721
**Smoking status**	Non-smoker versus current smoker	2.969	0.539	1.032	8.540	0.044

Covariates	Categories	HR	Standard error	Confidence interval (lower)	Confidence interval (upper)	P-value
	Past smoker versus non-smoker	4.056	0.542	1.403	11.731	0.010
**Sex**	Male versus female	1.726	0.248	1.061	2.808	0.028
**Socioeconomic status**	Medium versus low	0.944	0.259	0.569	1.567	0.823
	High versus low	0.000	3409.810	0.000	Inf	0.996
**Population sector**	Arab versus Orthodox Jewish	0.587	0.320	0.313	1.099	0.096
	Jewish versus Orthodox Jewish	1.189	0.399	0.543	2.601	0.665
**Immunosuppression**	Yes versus No	3.098	0.426	1.345	7.138	0.008
**Recent full vaccination**	Yes versus No	1.861	0.343	0.951	3.643	0.070
**Cardiovascular disease**	Yes versus No	2.233	0.248	1.374	3.628	0.001
**Diabetes**	Yes versus No	1.325	0.242	0.824	2.130	0.245
**Respiratory diseases**	Yes versus No	1.881	0.254	1.144	3.094	0.013
**Hypertension**	Yes versus No	1.596	0.272	0.936	2.721	0.086
**First vaccination dose**	0-3 weeks versus unvaccinated	0.200	0.295	0.112	0.357	0.000

Covariates	Categories	HR	Standard error	Confidence interval (lower)	Confidence interval (upper)	P-value
	4-7 weeks versus unvaccinated	0.196	0.434	0.084	0.459	0.000
	8-10 weeks versus unvaccinated	0.039	1.035	0.005	0.299	0.002
	11-19 weeks versus unvaccinated	0.000	3199.083	0.000	Inf	0.995
	≥ 20 weeks versus unvaccinated	0.357	0.806	0.074	1.734	0.202
**Chronic kidney disease**	Yes versus No	1.460	0.246	0.902	2.364	0.123
**Cancer**	Yes versus No	2.907	0.351	1.460	5.788	0.002
**Neurological disease**	Yes versus No	1.617	0.271	0.950	2.753	0.076
**Liver disease**	Yes versus No	0.847	0.522	0.304	2.358	0.750

Note: The Cox model was adjusted for age, population sector, sex, SES, BMI, number of flu vaccines received in the five years prior to COVID-19 infection, smoking status, recent full vaccination status, first vaccination dose, and chronic diseases (cancer, chronic kidney disease, respiratory diseases, cardiovascular diseases, diabetes, hypertension, immunosuppression, neurological conditions, and liver diseases). Two-sided *P*-value is reported.

Abbreviation: Inf: Infinite

Supplemental Table 3: Cox proportional hazard model for the association between REGEN-COV and risk for hospitalization

Covariates	Categories	HR	Standard error	Confidence interval (lower)	Confidence interval (upper)	P-value
**REGEN-COV (unadjusted)**	Yes versus no	0.632	0.276	0.368	1.086	0.097
**REGEN-COV (adjusted)**	Yes versus no	0.436	0.286	0.249	0.763	0.004
**Age**	Continuous	1.025	0.010	1.005	1.046	0.016
**Number of flu vaccination in the last five years**	1 versus 0	1.309	0.360	0.646	2.652	0.454
	2 versus 0	1.025	0.384	0.483	2.178	0.948
	3 versus 0	1.347	0.384	0.635	2.860	0.437
	4 versus 0	0.931	0.398	0.427	2.031	0.858
	5 versus 0	0.804	0.387	0.376	1.717	0.572
**Obesity**	Obese versus normal	1.827	0.272	1.072	3.115	0.027
	Overweight versus normal	1.465	0.281	0.845	2.540	0.174
	Underweight versus normal	1.015	0.793	0.215	4.800	0.985
**Smoking status**	Non-smoker versus current smoker	1.671	0.376	0.799	3.495	0.173
	Past smoker versus non-smoker	2.148	0.383	1.015	4.549	0.046

Covariates	Categories	HR	Standard error	Confidence interval (lower)	Confidence interval (upper)	P-value
**Sex**	Male versus female	1.372	0.213	0.903	2.085	0.138
**Socioeconomic status**	Medium versus low	1.108	0.226	0.712	1.724	0.651
	High versus low	0.000	2033.937	0.000	Inf	0.994
**Population sector**	Arab versus Orthodox Jewish	0.651	0.270	0.383	1.106	0.112
	Jewish versus Orthodox Jewish	1.099	0.357	0.546	2.213	0.791
**Immunosuppression**	Yes versus No	2.404	0.389	1.121	5.155	0.024
**Recent full vaccination**	Yes versus No	1.302	0.295	0.730	2.323	0.371
**Cardiovascular disease**	Yes versus No	1.926	0.222	1.247	2.974	0.003
**Diabetes**	Yes versus No	1.463	0.213	0.963	2.223	0.074
**Respiratory diseases**	Yes versus No	1.486	0.234	0.939	2.353	0.091
**Hypertension**	Yes versus No	1.758	0.239	1.101	2.808	0.018
**First vaccination dose**	0-3 weeks versus unvaccinated	0.165	0.270	0.097	0.281	0.000

Covariates	Categories	HR	Standard error	Confidence interval (lower)	Confidence interval (upper)	P-value
	4-7 weeks versus unvaccinated	0.232	0.365	0.114	0.475	0.000
	8-10 weeks versus unvaccinated	0.036	1.017	0.005	0.262	0.001
	11-19 weeks versus unvaccinated	0.258	0.620	0.077	0.868	0.029
	≥ 20 weeks versus unvaccinated	0.629	0.653	0.175	2.261	0.478
**Chronic kidney disease**	Yes versus No	1.623	0.214	1.068	2.468	0.023
**Cancer**	Yes versus No	2.252	0.318	1.208	4.197	0.011
**Neurological disease**	Yes versus No	1.670	0.242	1.039	2.685	0.034
**Liver disease**	Yes versus No	0.993	0.430	0.428	2.307	0.987

Note: The Cox model was adjusted for age, population sector, sex, SES, BMI, number of flu vaccines received in the five years prior to COVID-19 infection, smoking status, recent full vaccination status, first vaccination dose, and chronic diseases (cancer, chronic kidney disease, respiratory diseases, cardiovascular diseases, diabetes, hypertension, immunosuppression, neurological conditions, and liver diseases). Two-sided *P*-value is reported

Abbreviation: Inf: Infinite

Supplemental Table 4: Cox proportional hazard model for the association between REGEN-COV and risk for COVID-19 related death

Covariates	Categories	HR	Standard error	Confidence interval (lower)	Confidence interval (upper)	P-value
**REGEN-COV(unadjusted)**	Yes versus No	0.167	1.018	0.023	1.229	0.079
**REGEN-COV(adjusted)**	Yes versus No	0.065	1.019	0.009	0.479	0.007
**Age**	Continuous	1.107	0.021	1.061	1.154	0.000
**Number of flu vaccination in the last five years**	1 versus 0	14715265.761	0.543	5072758.428	42686646.626	0.000
	2 versus 0	32470083.242	0.462	13127755.157	80311240.810	0.000
	3 versus 0	4530811.907	0.735	1073762.042	19118068.740	0.000
	4 versus 0	8852132.913	0.419	3893051.401	20128235.936	0.000
	5 versus 0	7307643.966	0.420	3210173.099	16635134.210	0.000
**Obesity**	Obese versus normal	0.504	0.395	0.232	1.093	0.083
	Overweight versus normal	0.527	0.405	0.238	1.165	0.113
	Underweight versus normal	1.597	1.027	0.213	11.952	0.649

Covariates	Categories	HR	Standard error	Confidence interval (lower)	Confidence interval (upper)	P-value
**Smoking status**	Non-smoker versus current smoker	1.012	0.379	0.481	2.129	0.975
	Past smoker versus non-smoker	1.754	0.387	0.821	3.746	0.147
**Sex**	Male versus female	2.093	0.379	0.996	4.399	0.051
**Socioeconomic status**	Medium versus low	0.881	0.420	0.387	2.007	0.763
	High versus low	0.000	7886.083	0.000	Inf	0.998
**Population sector**	Arab versus Orthodox Jewish	0.239	0.735	0.057	1.007	0.051
	Jewish versus Orthodox Jewish	1.695	0.611	0.511	5.617	0.388
**Immunosuppression**	Yes versus No	0.000	5063.578	0.000	Inf	0.997
**Recent full vaccination**	Yes versus No	1.610	0.494	0.612	4.238	0.335
**Cardiovascular disease**	Yes versus No	1.949	0.388	0.910	4.173	0.086
**Diabetes**	Yes versus No	1.711	0.382	0.809	3.620	0.160
**Respiratory diseases**	Yes versus No	1.790	0.421	0.784	4.084	0.167

Covariates	Categories	HR	Standard error	Confidence interval (lower)	Confidence interval (upper)	P-value
**Hypertension**	Yes versus No	3.050	0.541	1.056	8.813	0.039
**First vaccination dose**	0-3 weeks versus unvaccinated	0.157	0.406	0.071	0.347	0.000
	4-7 weeks versus unvaccinated	0.244	0.612	0.074	0.811	0.021
	8-10 weeks versus unvaccinated	0.000	3640.551	0.000	Inf	0.996
	11-19 weeks versus unvaccinated	0.000	5945.661	0.000	Inf	0.997
	≥ 20 weeks versus unvaccinated	3.852	0.635	1.110	13.366	0.034
**Chronic kidney disease**	Yes versus No	1.097	0.395	0.506	2.378	0.814
**Cancer**	Yes versus No	1.831	0.504	0.682	4.912	0.230
**Neurological disease**	Yes versus No	1.426	0.407	0.642	3.168	0.383
**Liver disease**	Yes versus No	0.000	5505.635	0.000	Inf	0.997

Note: The Cox model was adjusted for age, population sector, sex, SES, BMI, number of flu vaccines received in the five years prior to COVID-19 infection, smoking status, recent full vaccination status, first vaccination dose, and chronic diseases (cancer, chronic kidney disease, respiratory diseases, cardiovascular diseases, diabetes, hypertension, immunosuppression, neurological conditions, and liver diseases). Two-sided *P*-value is reported

Abbreviation: Inf: Infinite

Supplemental Table 5: Outcomes associated with REGEN-COV treatment effectiveness

	REGEN-COV effectiveness 95%CI	
	**Age ≥60 years old**	**Age <60 years old**
Hospitalization due to COVID-19	57.0% (21.0%-76.4%)	91.5% (28.2%-99.0%)
Severe COVID-19	61.1% (20.3%-81.0%)	86.3% (-148%-99.2%)
Death due to COVID-19	94.4% (58.8%-99.2%)	NA

Note: Treatment effectiveness was measured as a 1-Hazard ratio (HR), derived from a Cox – proportional model that was applied after the matching. Patients were matched using propensity score matching, with the propensity score model including the following variables: Age, population sector, sex, SES, BMI, immunosuppression status, pregnancy, and first vaccination dose status.

The Cox model was then further adjusted for age, population sector, sex, SES, BMI, number of flu vaccines received in the five years prior to COVID-19 infection, smoking status, recent full vaccination status, first vaccination dose, and chronic diseases (cancer, chronic kidney disease, respiratory diseases, cardiovascular diseases, diabetes, hypertension, immunosuppression, neurological conditions, and liver diseases). Complete variable definitions are found in Supplemental Table 7.

Abbreviation: CI, confidence interval. NA, Not applicable due to rare outcome among the treated patients

Supplemental Table 6: Outcomes associated with REGEN-COV treatment effectiveness using a PSM approach

		Received REGEN-COV (n=289)	Did not received REGEN-COV (n=1,320)	Unadjusted REGEN-COV Effectiveness (95%CI)	Adjusted REGEN-COV effectiveness (95% CI
Hospitalization due to COVID-19	Yes	15	104	34.4% (-12.7%-61.8%)	44.2% (2.8%-68.0%)
Severe COVID-19	Yes	10	81	43.8% (-8.4%-70.9%)	50.2% (2.2%-74.7%)
Death due to COVID-19	Yes	1	26	82.2% (-30.9%-97.6%)	88.4% (8.7%-98.5%)

Note: Treatment effectiveness was measured as a 1-Hazard ratio (HR), derived from a Cox – proportional model that was applied after the matching. Patients were matched using propensity score matching, with the propensity score model including the following variables: Age, population sector, sex, SES, BMI, immunosuppression status, pregnancy, and first vaccination dose status.
The Cox model was then further adjusted for age, population sector, sex, SES, BMI, number of flu vaccines received in the five years prior to COVID-19 infection, smoking status, recent full vaccination status, first vaccination dose, and chronic diseases (cancer, chronic kidney disease, respiratory diseases, cardiovascular diseases, diabetes, hypertension, immunosuppression, neurological conditions, and liver diseases). Complete variable definitions are found in Supplemental Table 7.
Abbreviation: CI, Confidence Interval

Supplemental table 7: Variable defintion

Variable name	Defintion	Timing
Exposure	REGEN-COV (Yes/No)	September 19, 2021 - December 8, 2021
Outcome	Three different outcomes:<br><br>(1) Hospitalization due to COVID-19. Defined as hospitalization that was reported from the Israeli MOH as a hospitalization of a SARS-CoV-2 infected individuals.<br>(2) Severe COVID-19 was defined according to the Israeli Ministry of Health guidelines in a manner that is consistent with the NIH criteria for severe illness or critical illness. These definitions are: SpO2 (Oxygen saturation level) < 94% on room air at sea level, a ratio of arterial partial pressure of oxygen to fraction of inspired oxygen (PaO2/FiO2) <300 mm Hg, respiratory frequency >30 breaths/min, or lung infiltrates>50%. Furthermore, critical illness was included with severe COVID-19 and those who have respiratory failure, septic shock that may present virus-induced distributive shock, and/or multiple organ dysfunction syndrome or failure.[1]<br>(3) Death due to COVID-19. A death of a SARS-CoV-2 infected individual reported to the Israeli MOH.	(1) The start date of the hospitalization<br>(2) The first date during the hospitalization in which the individual was flagged as being in a severe or critical state<br>(3) The reported date of death

Age	Age at infection date. Defined as continuous and categorical.<br>Age group:<br>(1) 20-39; (2) 40-49; (3) 50-59; (4) 60-69; (5) 70-74; (6) ≥75	Current
Gender	Male/Female	Current
Socio-economic status	For matching – we used it as as a catigorical variable with 20 level. For table 1 we categorized it into 3 levels: Low, medium , and high	Current
Population group	Jewish, Arab, Ultra-Orthodox Jewish	Current
Flu vaccination in the last 5 years	Number of influenza vaccination in the last 5 yeas prior to the index date. It is categorical variable that ranges between 0 –doses up to at least 5 doses of flu vaccine in the last 5 years prior to the index date	Last 5 years
BMI	Body Mass Index :<br><br>Under weight <18.5<br>Normal weight –BMI: 18.5-25.0<br>Overweight- BMI: >25-30<br>Obesity – BMI: >30	Latest measurement in last 5 years not taken during pregnancy
Smoking status	Categorical variable. Current smoker, past smoker, non-smoker	
Recent full vaccination status	Defined as two or more vaccination doses, and fewer than 150 days from the most recent vaccination dose until the first positive PCR test(Yes/No)	
First vaccination dose	the number of weeks from the beginning of the COVID-19 national vaccination campaign until the first vaccination dose	
Hypertension	ICD9 Code 401*<br>ICD9 Code 402*<br>ICD9 Code 403*<br>ICD9 Code 404*<br>ICD9 Code 405*	Ever

Cardiovascular disease	ICD9 Code 410*<br>ICD9 Code 411*<br>ICD9 Code 412<br>ICD9 Code 413*<br>ICD9 Code 414*<br>ICD9 Code 429.2, 429.7*<br>ICD9 Code V45.81, V45.82<br>ICD9 Procedure Code 36.0*<br>ICD9 Procedure Code 36.1*<br>ICD9 Code 428*<br>ICD9 Code 398.91<br>ICD9 Code 402._1<br>ICD9 Code 404._1, ICD9 Code 404._3<br>ICD9 Code 416.9<br>ICD9 Code 514<br>ICD9 Code 425*<br>ICD9 Code 416*	Ever
Respiratory disease	Having any pulmonary disease (mentioned below) prior to the index date<br>COPD: ICD9 codes: 491.2*, 492.* 496.*<br>Asthma: ICD9 code: 493.*<br>Other respiratory disease: ICD9 Code 277.0* ICD9: Code 494*. ICD9 : Code 515.*	Ever
Neurological conditions	ICD9 Code 290.*<br>ICD9 Code 294*<br>ICD9 Code 310.1<br>ICD9 Code 331*<br>ATC Codes N06DA02, N06DA03<br>ICD9 Code 358*<br>ICD9 Code 332.[0,1]<br>ICD9 Code 345*<br>ICD9 Code 340<br>ATC Codes L03AB07, L03AB08, L04AA07<br>ICD9 Code 343*<br>ICD9 Code 333.4<br>ICD9 Code 334*<br>ICD9 Code 356*<br>ICD9 Code 138<br>ICD9 Code 335*<br>ICD9 Code 730.7*<br>ICD9 V12.02	For diagnosis codes, Ever For drugs, 4 or more dispensed in last 12 months

	ICD9 Code 228.02<br>ICD9 Code 307.23<br>ICD9 Code 330.9<br>ICD9 Code 331.3*<br>ICD9 Code 331.4<br>ICD9 Code 333*<br>ICD9 Code 334*<br>ICD9 Code 336*<br>ICD9 Code 337<br>ICD9 Code 335.1*<br>ICD9 Code 359.0<br>ICD9 Code 359.21<br>ICD9 Code 357.0<br>ICD9 Code 237.7*<br>ICD9 Code 742.8[1,2]	
Cancer	ICD9 Code 174*<br>ICD9 Code 175*<br>ICD9 Code 233.0<br>ICD9 Code V10.3<br>ICD9 Procedure Code 85.4*<br>ICD9 Code 153*<br>ICD9 Code 154*<br>ICD9 Code V10.5*<br>ICD9 Code V10.6*<br>ICD9 Code 185<br>ICD9 Code V10.46<br>ICD9 Code 162*<br>ICD9 Code V10.1*<br>ICD9 Code 188*<br>ICD9 Code V10.51<br>ICD9 Code 183*<br>ICD9 Code V10.43<br>ICD9 Code 179<br>ICD9 Code 182*<br>ICD9 Code V10.42<br>ICD9 Code 157*<br>ICD9 Code 191*<br>ICD9 Code 192*<br>ICD9 Code V10.85<br>ICD9 Code 151*<br>ICD9 Code V10.04<br>ICD9 Code 172*<br>ICD9 Code V10.82<br>ICD9 Code 201*<br>ICD9 Code 200*<br>ICD9 Code 202.4*<br>ICD9 Code 204*	Last 5 years

	ICD9 Code 205*<br>ICD9 Code 206*<br>ICD9 Code 207.1*<br>ICD9 Code 208.1*<br>ICD9 Code 189*<br>ICD9 Code V10.52<br>ICD9 Code 160*<br>ICD9 Code 161*<br>ICD9 Code 164.0<br>ICD9 Code 195.0<br>ICD9 Code V10.21<br>ICD9 Code V10.22<br>ICD9 Code 180*<br>ICD9 Code V10.41<br>ICD9 Code 140*<br>ICD9 Code 141*<br>ICD9 Code 142*<br>ICD9 Code 143*<br>ICD9 Code 144*<br>ICD9 Code 145*<br>ICD9 Code 150*<br>ICD9 Code V10.03<br>ICD9 Code 155*<br>ICD9 Code 156*<br>ICD9 Code V10.07<br>ICD9 Code 170*<br>ICD9 Code V10.81<br>ICD9 Code 193<br>ICD9 Code V10.87<br>ICD9 Code 171*<br>ICD9 Code 176*<br>ICD9 Code 184*<br>ICD9 Code 186*<br>ICD9 Code 187*<br>ICD9 Code V10.4*<br>ICD9 Code 203*<br>ICD9 Code 273.3<br>ICD9 Code 152*<br>ICD9 Code 158*<br>ICD9 Code 159*<br>ICD9 Code 163*<br>ICD9 Code 164*<br>ICD9 Code 165*<br>ICD9 Code 181<br>ICD9 Code 190*<br>ICD9 Code 192.8<br>ICD9 Code 196*	

	ICD9 Code 197*<br>ICD9 Code 198*	
Chronic kidney disease	ICD Procedure Code 39.95<br>ICD Procedure Code 54.98<br>ICD9 Code 996.81<br>ICD9 Code V42.0<br>ICD Procedure Code 55.6*<br>ICD9 Code 403._1<br>ICD9 Code 404._2<br>ICD9 Code 404._3<br>ICD9 Code 585*<br>ICD9 Code 586<br>ICD9 Code 250.4*<br>ICD9 Code 274.1*<br>ICD9 Code 440.1<br>ICD9 Code 581*<br>ICD9 Code 582*<br>ICD9 Code 583*<br>ICD9 Code 587<br>ICD9 Code 588*<br>ICD9 Code 589*	Ever
Pregnancy	Internal Clalit Registry	Current
Diabetes	Having type 2 Diabetes or having Type 1 diabetes.<br>Type 2 diabetes (T2DM):<br>HbA1C > 6.5<br>ATC Codes A10[A,B]<br>ICD9 Code 250*<br>ICD9 Code 357.2<br>ICD9 Code 362.0*<br>And not:<br>ICD9 Code 250._1, 250._3<br><br>Type 1 diabetes (T1DM):<br>ICD9 Code 250._1, 250._3	T2DM: For diagnosis codes, Ever For drugs, 4 or more dispensed in last 12 months<br><br>T1DM:<br>Ever
Hypertension	ICD9 Code 401*<br>ICD9 Code 402*<br>ICD9 Code 403*<br>ICD9 Code 404*<br>ICD9 Code 405*	Ever
Immunosuppression	Any of:<br>ICD9 Code 042*<br>ICD9 Code 043*<br>ICD9 Code 044*<br>ICD9 Code 795.71<br>ICD9 Code V08<br>ICD9 Code V42.8*	For diagnosis codes, Ever<br>For drugs, 4 or more dispensed in last 12 months

	ICD9 Proc Code 41.0*<br>Or at least 2 of:<br>ATC4 Code H02AB<br>ATC4 Code H02BX<br>ATC4 Code M01BA<br>Or at least 2 of:<br>ATC2 Code L04	
Liver disease	ICD9 Code 070.22<br>ICD9 Code 070.23<br>ICD9 Code 070.32<br>ICD9 Code 070.33<br>ICD9 Code 070.44<br>ICD9 Code 070.54<br>ICD9 Code V02.61<br>ICD9 Code V02.62<br>ICD9 Code 571*<br>ICD9 Code 275.1<br>ICD9 Code 277.4<br>ICD9 Code 452<br>ICD9 Code 453.0<br>ICD9 Code 571.8<br>ICD9 Code 571.9	Ever

Note: Additional confirmation of the diagnostic codes was done by checking the matching of the free text within the diagnosis description field.

Abbreviations: CHS: Clalit Health Services; ICD, International Classification of Disease. ATC, Anatomic therapeutic chemical

Supplemental Figure legends:

Supplemental figure 1: Flow chart for study participants.
Supplemental Figure 1 describes the process of the participants that meets the inclusion – exclusion criteria for the study

Supplemental Figure 2: Love Plot for the Covariate Balance.
A covariate balance (Love) plot for the matched and unmatched, showing the difference in means for different set of covariates (confounders). It was assessed by absolute mean difference. A strict balance cut-off was set at 0.1.

Supplemental figure 1

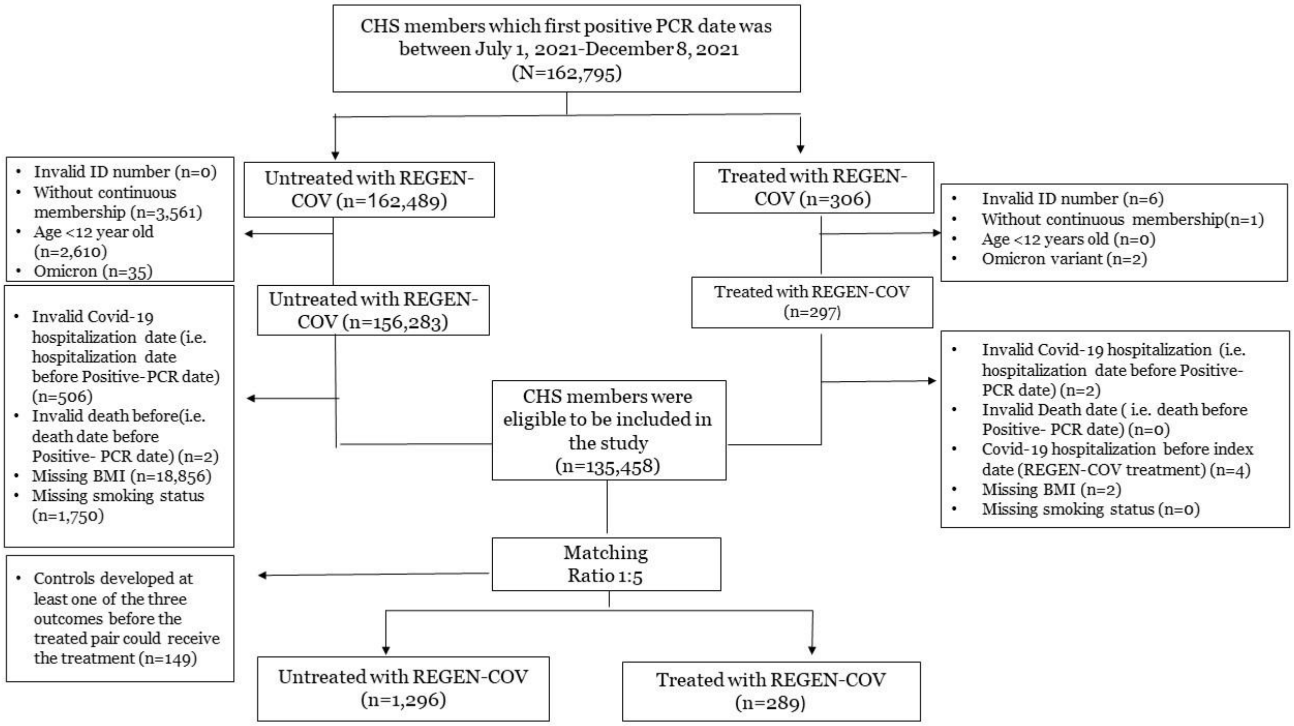

Supplemental Figure 2.

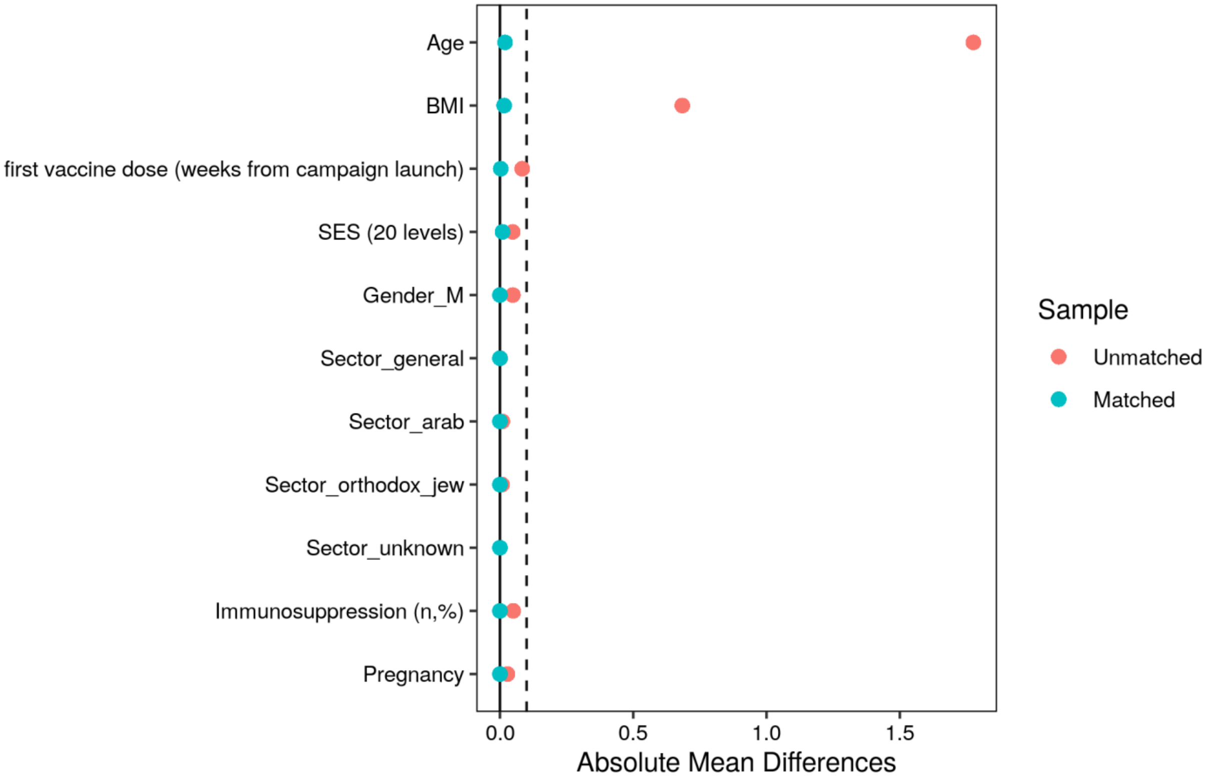

Supplement: Supplementary file 1 — Supplementary Information [file 41467_2022_32253_MOESM1_ESM.pdf]
