# Supplementary material for: Effectiveness of REGEN-COV antibody combination in preventing severe COVID-19 outcomes
Source: Nat Commun. 2022 Aug 2;13:4480. doi: 10.1038/s41467-022-32253-9 (PMC9344792; doi:10.1038/s41467-022-32253-9)
Supplement: Supplementary file 2 — Reporting Summary [file 41467_2022_32253_MOESM2_ESM.pdf]

nature portfolio

Corresponding author(s): Samah Hayek

Last updated by author(s): 2022/07/13

Reporting Summary

Nature Portfolio wishes to improve the reproducibility of the work that we publish. This form provides structure for consistency and transparency in reporting. For further information on Nature Portfolio policies, see our Editorial Policies and the Editorial Policy Checklist.

Statistics

For all statistical analyses, confirm that the following items are present in the figure legend, table legend, main text, or Methods section.

n/a	Confirmed	
☐	☒	The exact sample size (*n*) for each experimental group/condition, given as a discrete number and unit of measurement
☐	☒	A statement on whether measurements were taken from distinct samples or whether the same sample was measured repeatedly
☐	☒	The statistical test(s) used AND whether they are one- or two-sided<br>*Only common tests should be described solely by name; describe more complex techniques in the Methods section.*
☐	☒	A description of all covariates tested
☐	☒	A description of any assumptions or corrections, such as tests of normality and adjustment for multiple comparisons
☐	☒	A full description of the statistical parameters including central tendency (e.g. means) or other basic estimates (e.g. regression coefficient) AND variation (e.g. standard deviation) or associated estimates of uncertainty (e.g. confidence intervals)
☐	☒	For null hypothesis testing, the test statistic (e.g. *F*, *t*, *r*) with confidence intervals, effect sizes, degrees of freedom and *P* value noted<br>*Give P values as exact values whenever suitable.*
☒	☐	For Bayesian analysis, information on the choice of priors and Markov chain Monte Carlo settings
☒	☐	For hierarchical and complex designs, identification of the appropriate level for tests and full reporting of outcomes
☒	☐	Estimates of effect sizes (e.g. Cohen's *d*, Pearson's *r*), indicating how they were calculated

Our web collection on statistics for biologists contains articles on many of the points above.

Software and code

Policy information about availability of computer code

Data collection	Data were extracted from CHS electronic medical records. Data were extracted using SQL 2017.
Data analysis	The statistical analyses were performed using R version 3.5.2

For manuscripts utilizing custom algorithms or software that are central to the research but not yet described in published literature, software must be made available to editors and reviewers. We strongly encourage code deposition in a community repository (e.g. GitHub). See the Nature Portfolio guidelines for submitting code & software for further information.

Data

Policy information about availability of data

All manuscripts must include a data availability statement. This statement should provide the following information, where applicable:

- Accession codes, unique identifiers, or web links for publicly available datasets
- A description of any restrictions on data availability
- For clinical datasets or third party data, please ensure that the statement adheres to our policy

Provide your data availability statement here.

Human research participants

Policy information about studies involving human research participants and Sex and Gender in Research.

Reporting on sex and gender	We used the term "sex" in our analysis Data
Population characteristics	We conducted a cohort study using CHS data to estimate the real-world effectiveness of REGEN-COV in preventing severe COVID-19-related outcomes. Eligibility criteria included: a documented first positive SARS-CoV-2 polymerase-chain-reaction (PCR) test result; a determination of being high risk for severe COVID-19 based on medical history and clinical characteristics; age 12 years-old or older; and at least one year of continuous CHS membership as of the infection date. We excluded patients who were known to be infected with the Omicron variant. We assessed Omicron infections based on sequencing of viral samples or the S-gene target failure (SGTF) technique; the prevalence of Omicron during the study period was negligible. We also excluded patients with invalid outcome data (e.g. invalid hospitalization data, etc) and those who received a positive PCR result during hospitalization for another condition.
Recruitment	Data were extracted from CHS electronic medical records
Ethics oversight	The study was approved by CHS institutional review board (0052-20-COM2)

Note that full information on the approval of the study protocol must also be provided in the manuscript.

Field-specific reporting

Please select the one below that is the best fit for your research. If you are not sure, read the appropriate sections before making your selection.

☐ Life sciences ☒ Behavioural & social sciences ☐ Ecological, evolutionary & environmental sciences

For a reference copy of the document with all sections, see nature.com/documents/nr-reporting-summary-flat.pdf

Behavioural & social sciences study design

All studies must disclose on these points even when the disclosure is negative.

Study description	We conducted a cohort study using CHS data to estimate the real-world effectiveness of REGEN-COV in preventing severe COVID-19-related outcomes. Eligibility criteria included: a documented first positive SARS-CoV-2 polymerase-chain-reaction (PCR) test result; a determination of being high risk for severe COVID-19 based on medical history and clinical characteristics; age 12 years-old or older; and at least one year of continuous CHS membership as of the infection date. We excluded patients who were known to be infected with the Omicron variant. We assessed Omicron infections based on sequencing of viral samples or the S-gene target failure (SGTF) technique; the prevalence of Omicron during the study period was negligible. We also excluded patients with invalid outcome data (e.g. invalid hospitalization data, etc) and those who received a positive PCR result during hospitalization for another condition.<br>To emulate a target trial, treated patients were individually matched with non-treated patients. Treated patients were those with a first positive PCR test result obtained between September 19, 2021 and December 8, 2021 and who received REGEN-COV treatment: Non-treated patients were those who obtained a first positive PCR test result between July 1, 2021 and December 8, 2021 and who did not receive REGEN-COV treatment. The recruitment period for the non-treated patients was a few weeks longer than for the treated patients in order to increase the sample size of the non-treated group and allow for a 1:5 matching of treated to non-treated individuals. Matching was performed using an optimal matching scheme. The Mahalanobis distance metric was used for continuous variables, and exact matching was used for categorical variables.16,17 Optimal matching minimizes the overall pairwise distances without dependency on the order of matching.
Research sample	Data was extracted from the CHS database. CHS is the largest integrated payer-provider healthcare organization in Israel. The CHS database contains extensive medical histories of CHS's 4.7 million members, including COVID-19 test results and outcomes. These data repositories have been previously described in detail.<br>A retrospective cohort study was conducted using the data repositories of Israel's largest healthcare organization. Patients diagnosed with COVID-19 between September 19, 2021, and December 8, 2021, who were treated with REGEN-COV were matched to patients diagnosed with COVID-19 between July 1, 2021 and December 8, 2021 who were not treated with REGEN-COV.
Sampling strategy	A retrospective cohort study was conducted using the data repositories of Israel's largest healthcare organization. Patients diagnosed with COVID-19 between September 19, 2021, and December 8, 2021, who were treated with REGEN-COV were matched to patients diagnosed with COVID-19 between July 1, 2021 and December 8, 2021 who were not treated with REGEN-COV.<br><br>Demographic and clinical characteristics were used to match patients and for further adjustment as part of the Cox model.<br><br>In total, 162,795 CHS members tested positive for COVID-19 between July 1 and December 8, 2021. Of this population, 306 were enrolled in our cohort and treated with REGEN-COV between September 19, 2021 and December 8, 2021, and 162,489 were non-treated with REGEN-COV. Exclusion criteria for both treated and non-treated with REGEN-COV included: participants aged <12 years old (n=2,610); participants without continuous CHS membership (n=3,562); participants who previously had the omicron variant

	(n=37); participant data that had invalid information related to COVID-19 (e.g., death date, hospitalization date) (n=514); participants hospitalized before the index date(n=4); missing data on participant smoking status (n=1,750), and missing data on BMI (n=18,857). 135,458 were eligible for inclusion in the study, and patients were matched on a 1:5 ratio. Of those, 149 controls were excluded before contributing follow-up time as they developed one of the outcomes before their treated match could receive the treatment. The final analysis included 289 patients treated with REGEN-COV and 1,296 non-treated matched patients.(Supplemental Figure 1)
Data collection	Data was extracted from CHS electronic medical record.
Timing	For treated patients we included participants that had positive PCR between September 19,2021 - December 8, 2021. For control group, we included those who had positive PCR between July 1, 2021- December 8, 2021.
Data exclusions	We excluded patients who were known to be infected with the Omicron variant. We assessed Omicron infections based on sequencing of viral samples or the S-gene target failure (SGTF) technique; the prevalence of Omicron during the study period was negligible. We also excluded patients with invalid outcome data (e.g. invalid hospitalization data, etc) and those who received a positive PCR result during hospitalization for another condition.
Non-participation	Unmatched non- treated participants (N=133,873)
Randomization	It is not a clinical trial, so we did not have any randomization. However, we emulate target trial and we used matching to control for the confounders.

Reporting for specific materials, systems and methods

We require information from authors about some types of materials, experimental systems and methods used in many studies. Here, indicate whether each material, system or method listed is relevant to your study. If you are not sure if a list item applies to your research, read the appropriate section before selecting a response.

Materials & experimental systems

n/a	Involved in the study
☒	☐ Antibodies
☒	☐ Eukaryotic cell lines
☒	☐ Palaeontology and archaeology
☒	☐ Animals and other organisms
☒	☐ Clinical data
☒	☐ Dual use research of concern

Methods

n/a	Involved in the study
☒	☐ ChIP-seq
☒	☐ Flow cytometry
☒	☐ MRI-based neuroimaging
